# Two-Dimensional Covalent Organic Frameworks: Structural Insights across Different Length Scales and Their Impact on Photocatalytic Efficiency

**DOI:** 10.1021/acs.accounts.4c00491

**Published:** 2024-10-22

**Authors:** Islam E. Khalil, Prasenjit Das, Arne Thomas

**Affiliations:** † Department of Chemistry, Functional Materials Technische Universität Berlin, 10623 Berlin, Germany

## Abstract

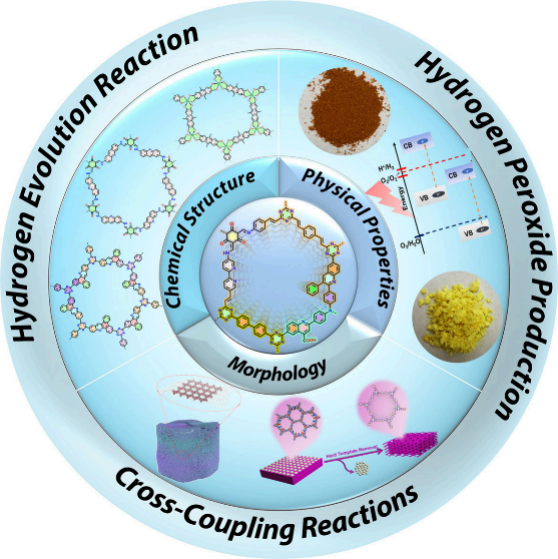

Covalent organic frameworks (COFs) are a rapidly emerging class of crystalline porous polymers, characterized by their highly defined, predictable, and tunable structure, porosity, and properties. COFs can form both two-dimensional (2D) and three-dimensional (3D) architectures, each with unique characteristics and potential applications. 2D COFs have attracted particular interest due to their favorable structural and optoelectronic properties. They can be equipped with a range of different functional moieties in their backbone, ranging from acidic to basic, from hydrophilic to hydrophobic, and from metal-coordinating to redox-active functions. In addition, their crystallinity, high specific surface area, and remarkable thermal and chemical stability make them attractive for a variety of applications, including gas separation, catalysis, energy storage, and optoelectronics.

This Account provides a detailed overview of our recent efforts to synthesize and apply 2D COFs. First, various synthesis routes are discussed, focusing on methods that involve reversible and irreversible linkage reactions. Reversible reactions, such as imine or boronate ester formation, are advantageous for producing highly crystalline COFs because they allow for error correction during synthesis. In contrast, irreversible reactions, such as carbon–carbon or carbon–nitrogen bond formation, yield COFs with greater chemical stability, although controlling crystallinity can be more challenging. Our group has contributed significantly to refining these methods to balance crystallinity and stability, enhancing the performance of the resulting 2D COFs.

In addition to different binding patterns, we have also developed strategies to control the micro- and macromorphologies of COFs, which is crucial for optimizing their properties for specific applications. For example, we have explored the synthesis of hierarchical porous COFs by using templating techniques or by forming composites with other functional materials. These strategies enable us to fine-tune the porosity and surface properties of COFs, thereby improving their performance in applications like catalysis. Hierarchical structures in particular enhance photocatalytic efficiency by providing a larger surface area for light absorption and facilitating the transport of photogenerated charge carriers.

We further examine the practical applications of 2D COFs, with a primary focus on photocatalysis. Photocatalysis uses light to enable or accelerate chemical reactions, and 2D COFs are ideal for this purpose due to their tunable band gaps and large surface areas. Our research has shown that 2D COFs are highly versatile photocatalysts that can effectively catalyze reactions such as water splitting, carbon dioxide reduction, hydrogen peroxide formation, and cross-coupling reactions. By exploiting the unique properties of 2D COFs, we have achieved significant improvement in many photocatalytic reactions.

With this comprehensive overview, we aim to contribute to the further development and understanding of 2D COFs and encourage further research and innovation in this promising field.

## Key References

- LiC.; YangJ.; PachfuleP.; LiS.; YeM.-Y.; SchmidtJ.; ThomasA.Ultralight covalent organic framework/graphene aerogels with hierarchical porosity. Nat. Commun.2020, 11, 471232948768 10.1038/s41467-020-18427-3PMC7501297.^1^ A COF/rGO composite possesses high mechanical strength, withstanding repeated compression. Its graphene network ensures efficient electron transport, enabling use as a supercapacitor electrode without binders, offering superior capacitance and enhanced cycling stability.
- YangJ.; AcharjyaA.; YeM. Y.; RabeahJ.; LiS.; KochovskiZ.; YoukS.; RoeserJ.; GrunebergJ.; PenschkeC.; SchwarzeM.; WangT.; LuY.; van de KrolR.; OschatzM.; SchomackerR.; SaalfrankP.; ThomasA.Protonated Imine-Linked Covalent Organic Frameworks for Photocatalytic Hydrogen Evolution. Angew. Chem., Int. Ed.2021, 60, 19797–1980310.1002/anie.202104870PMC845721034043858.^2^ Protonating imine-linked COFs enhances their optoelectronic properties, significantly boosting photocatalytic hydrogen evolution through improved light absorption, charge separation, and hydrophilicity, leading to a marked increase in performance.
- YangJ.; GhoshS.; RoeserJ.; AcharjyaA.; PenschkeC.; TsutsuiY.; RabeahJ.; WangT.; Djoko TameuS. Y.; YeM.-Y.; GrunebergJ.; LiS.; LiC.; SchomackerR.; Van De KrolR.; SekiS.; SaalfrankP.; ThomasA.Constitutional isomerism of the linkages in donor-acceptor covalent organic frameworks and its impact on photocatalysis. Nat. Commun.2022, 13, 631736274186 10.1038/s41467-022-33875-9PMC9588771.^3^ Constitutional isomers of imine-linked donor–acceptor (D-A) COFs were synthesized and exhibited significant differences in their photophysical properties and photocatalytic efficiency.
- DasP.; ChakrabortyG.; FrieseN.; RoeserJ.; PrinzC.; EmmerlingF.; SchmidtJ.; ThomasA.Heteropolyaromatic Covalent Organic Frameworks via One-Pot Multicomponent Reactions. J. Am. Chem. Soc.2024, 146, 17131–1713938875002 10.1021/jacs.4c02551PMC11212053.^4^ We introduce the first structural isomeric COFs produced via multicomponent domino and Povarov reactions utilizing epoxystyrene to synthesize unique 2,3-phenylquinoline COFs. Additionally, the 2,3-phenylquinolines can undergo a Scholl reaction to form extended aromatic linkages.
- KhalilI. E.; DasP.; Küçükkeçeci, H.; Dippold, V.; Rabeah, J.; Tahir, W.; Roeser, J.; SchmidtJ.; ThomasA.Hierarchical Porous Covalent Organic Frameworks: The Influence of Additional Macropores on Photocatalytic Hydrogen Evolution and Hydrogen Peroxide Production. Chem. Mater.2024, 368330–8337. We utilized polystyrene spheres (PSs) as hard templates to generate interconnected macropores in an otherwise only microporous COF. This macro/microporous COF promotes faster mass transport and improves HER efficiency and H_2_O_2_ production by enhancing active site accessibility.

## 1 Introduction

The exploration of two-dimensional (2D) materials gained substantial momentum following the discovery of graphene, layered transition metal oxides, and chalcogenides or MXenes.^5^ Consequently, materials such as black phosphorus, boron nitride, and layered oxides and hydroxides from group IV elements have attracted significant attention.^6^ Covalent organic frameworks (COFs) can form organic, porous, and 2D layered materials. The organic nature of COFs offers the advantage of a modular design and thus the possibility of tailoring their structures to implement specific properties.^7−9^ COFs are characterized by the covalent linkages between organic building blocks, forming predictable and periodic two- or three-dimensional (3D) networks.^10^ This structural design permits rational control over the chemical composition and topology of COFs through careful selection of monomers.^11^ Furthermore, COFs feature uniform nanometer-scale pores whose shapes, dimensions, and functionalization are governed by the monomer structure. These pores enable the rapid transport of ions or molecules through the material and thus access to functional groups of the framework material. In addition, COFs offer unique advantages such as low density, π-conjugated structures, and high structural stability due to covalent bonds. These properties make COFs promising materials for various applications.^9^

While early COFs suffered from water instability, a number of advancements have produced COFs which are resilient even in harsh chemical environments.^12,13^ While recent reviews have focused on the general synthesis and applications of 2D COFs, in this Account, we highlight our own recent efforts on 2D COFs and our contribution to the creation of stable, crystalline 2D COFs.

## 2 Synthesis of 2D COFs

### 2.1 General Methods for 2D COF Synthesis

**Scheme 1:**
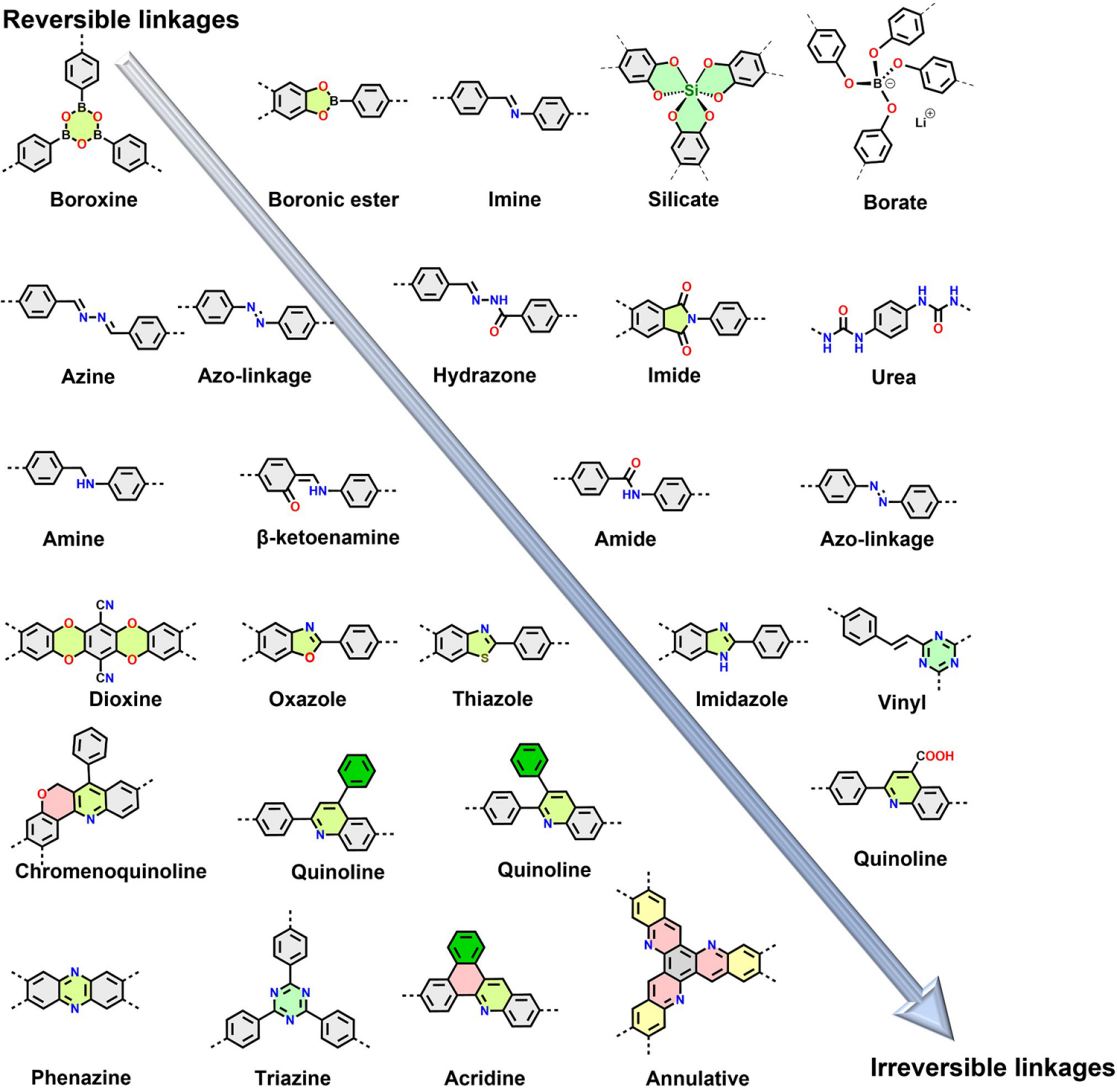
Linkages Used for Constructing COFs, Starting from Highly Reversible Linkages to Irreversible Linkages

The first syntheses of COFs involved reversible polycondensation reactions employing various linkages such as boroxines, boronic esters, imines, squaraines, or azines. However, other polycondensation reactions, particularly those involving phenazine and C=C linkages, have gained prominence due to their enhanced stability and extended π-conjugation (Scheme 1). The robustness of COFs is based on the covalent chemical bonds, whereby in most cases, sp^2^ hybridization of all backbone atoms is obtained, yielding an extended π-conjugation. Guided by topology diagrams, these polycondensation reactions facilitate the construction of either 2D or 3D polygonal networks. In 2D COFs, polymer sheets extend over the *xy*-plane and stack along the *z*-direction, driven by π–π interactions. The formation of the final framework yields rapid precipitation, resulting in aggregated polycrystalline materials. This poses challenges in obtaining isolated single crystals of 2D COFs, as modulating polycondensation, π–π interactions, crystallization, and precipitation within a one-pot system proves challenging due to the divergent nature of these processes.^7,14^ In this section, we categorize the so far reported linkages into reversible and (quasi-)irreversible types.

#### 2.1.1 Reversible Linkages

**Figure 1:**
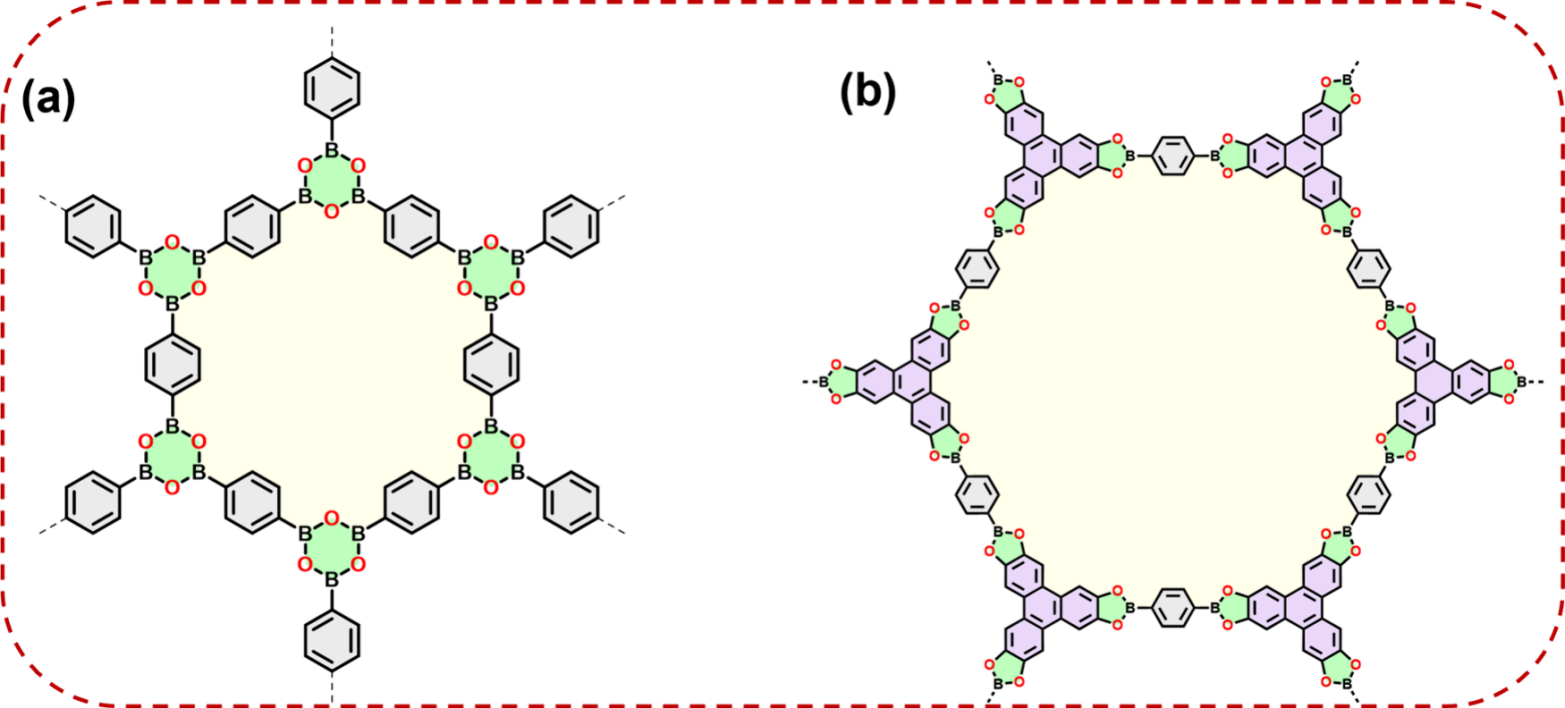
(a, b) Chemical structures of one layer of (a) COF-1 and (b) COF-5. Adapted from ref (10). Copyright 2005 AAAS.

The conventional strategy for engineering crystalline COFs utilizes dynamic covalent chemistry (DCC), i.e., the formation of reversible covalent bonds with control over the chemical equilibrium. This reversible bonding mechanism facilitates the assembly of building blocks into a thermodynamically favorable configuration. Any deviation from a crystalline structure or the formation of defects is energetically unfavorable and is thus repaired by following the DCC approach. In 2005, Yaghi and his team achieved a significant breakthrough with the synthesis of the first COFs, COF-1 and COF-5,^10^ which involved the self-condensation of boronic acid and the condensation of boronic acid with hydroxyl groups (Figure 1a,b). The resulting COF structure comprised boroxine rings and boronate ester linkages, exhibiting remarkable crystallinity facilitated by the reversible nature of these reactions. COFs containing these linkages were extensively studied during the early stages of COF material exploration.

Research in Schiff-base imine-linked COFs began with the discovery of COF-300 again by Yaghi and colleagues.^15^ This breakthrough was achieved through a solvothermal process involving terephthaldehyde and tetra-(4-anilyl)methane, catalyzed by aqueous acetic acid (AcOH), leading to the formation of reversible imine linkages. The resulting Schiff-base (imine-linked) COFs, when compared to their counterparts formed via B–O linkages, exhibit superior structural stability in various environments, including water, traditional organic solvents, and solutions with varying levels of acidity or alkalinity. Furthermore, these COFs display enhanced π-conjugation throughout their structure, facilitating efficient charge transport across the entire framework.^11^ Since then, a large number of imine-containing 2D COF with tailorable properties have been explored.^15^

The stabilization of imine linkages was achieved through the incorporation of an ortho hydroxy group onto the aldehyde moiety. This modification induces keto–enol tautomerism and facilitates intramolecular hydrogen bonding, thereby enhancing the stability of the bond.^13^ The number of imine linkages vs β-ketoenamine linkages formed via keto–enol tautomerization can be fine-tuned by the number of hydroxy groups present in the 1,3,5-benzene trialdehyde linker. Indeed, several groups have investigated the change of properties by continuously changing from COFs entirely formed from β-ketoenamine to fully imine-linked COFs as boundary cases.^16^ As example, we linked a diaminoacridine dye to such aldehyde linkers, obtaining four different COFs with changing optical properties and different activities when used as photosensitizer in metallaphotocatalytic cross-coupling reactions.^17,18^

It should be noted that new results question how universally the concept of DCC, including reversible bonding and bond breaking, can be applied to COF formation. First, novel reaction processes for imines and other COFs have recently been presented that actually seem not to allow extensive error correction, e.g., precipitation of COFs from aqueous solutions.^19^ Such reaction pathways, which do not require closed quartz ampules, complicated organic solvent mixtures, and high temperatures, are of course a stroke of luck, as they enable sustainable and, above all, scalable COF syntheses for the first time. Second, in recent years, new types of linkages have been introduced that are so chemically robust that they can be regarded as irreversible.

#### 2.1.2 (Quasi-)Irreversible Linkages

**Scheme 2:**
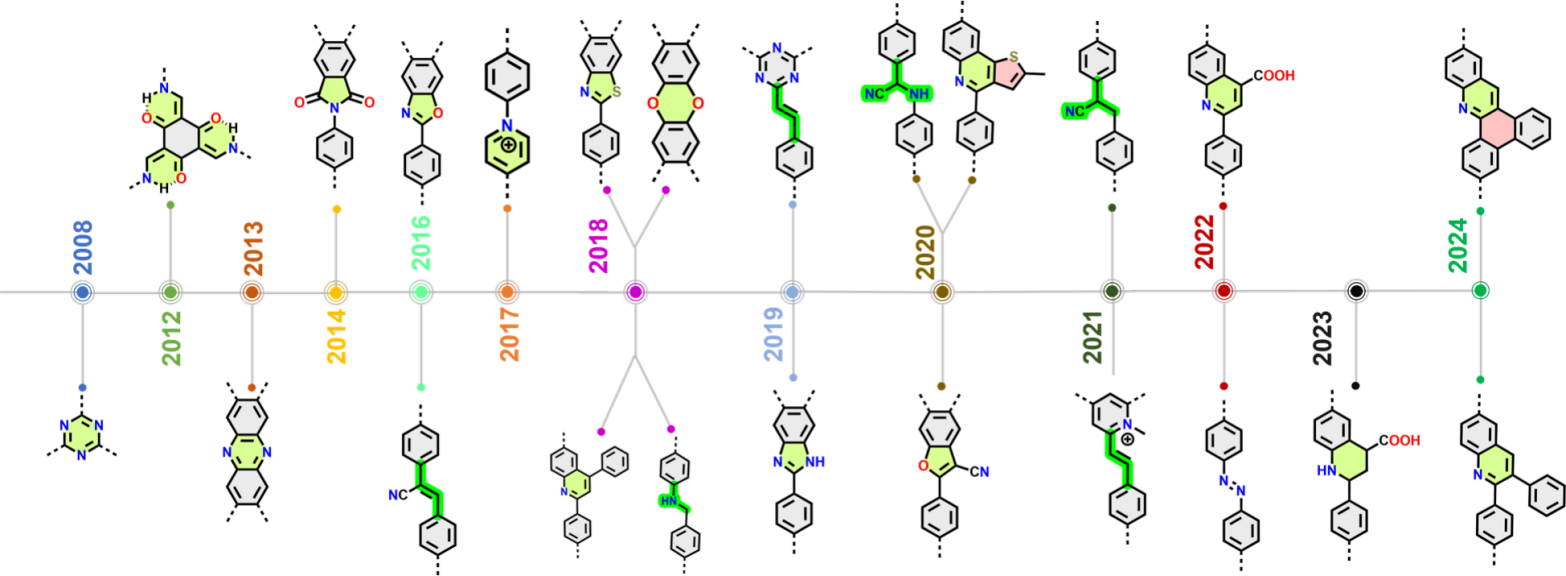
Timeline of Chemically Robust Linkers Formed via (Quasi-)Irreversible Chemical Transformation for the Construction of COFs

Besides ionic and metallic bonds, the covalent bond is assigned to strong chemical bonds, with binding energies ranging from 100 to 1000 kJ/mol. Hence, covalently bonded materials are expected to possess high chemical stability. Prior to the discovery of the concept of DCC, covalent bond formations were in general regarded as irreversible reactions.^20^ Irreversible chemical reactions can be defined as unidirectional processes, where the reactants convert to products but the products cannot convert back to the reactants or any other product under the particular reaction condition.^20^ It should be, however, noted that from thermodynamic principles, any chemical reaction is an equilibrium reaction (thus in principle reversible) and can just be regarded as “irreversible” when the equilibrium is far (actually entirely) on one side of the reaction because of the kinetic barrier and thermodynamic stability of the products and/or when a product or byproduct is removed (and thus no longer available for the back reaction), following Le Chatelier’s principle. Many covalent bond formations are thus essentially irreversible due to the high bond energies of the formed product, and very stable side products are often formed, which are removed from the equilibrium (e.g., as seen in cross-coupling reactions). Therefore, it seemed implausible for a long time that extended crystalline materials can be produced via covalent bond formations, that is, under thermodynamic control, as such irreversible reactions would lead to the kinetic, i.e., amorphous, product, as exemplified in the plethora of amorphous polymer networks.^21^ Here, we therefore mainly concentrate on reactions that form COFs with strong covalent bonds (from a thermodynamic and kinetic viewpoint), that is, those that possess high chemical stability and would be regarded as irreversible when applied in conventional organic or polymer synthesis (Scheme 2).

**Figure 2:**
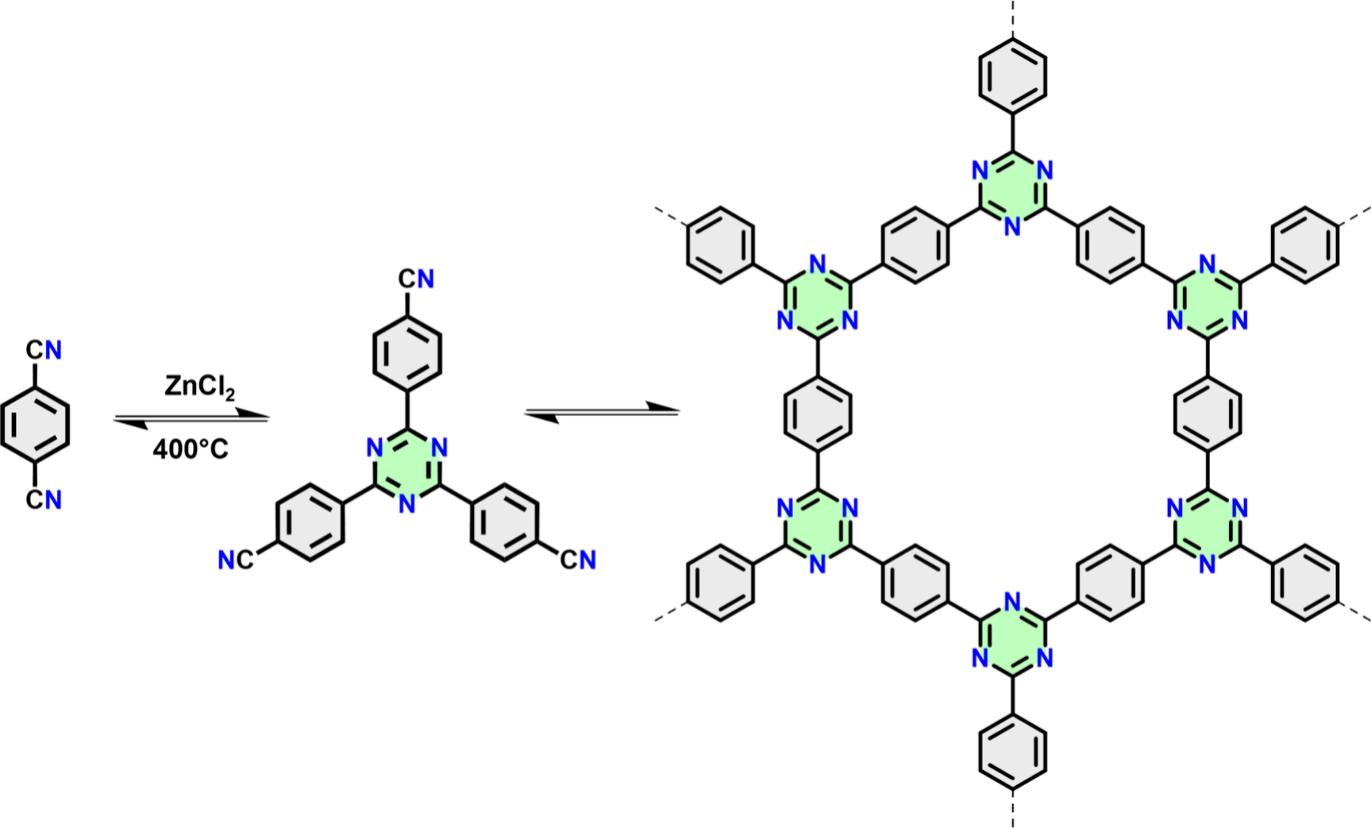
Trimerization of dicyanobenzene in molten ZnCl_2_ to trimers and oligomers and then to CTF-1. Adapted from ref (22). Copyright 2008 Wiley-VCH.

To enhance the chemical stability of COFs, in 2008 an ionothermal synthesis of crystalline covalent triazine frameworks (CTFs) was reported, where different types of aromatic nitriles are cyclotrimerized in the presence ZnCl_2_ at elevated temperature (400 °C) (Figure 2).^22^ It was mentioned that such harsh reaction conditions and the Lewis-acidic catalytic activity of ZnCl_2_ were needed to ensure the reversibility of the triazine forming reaction (which, as mentioned, questions the term irreversibility in this context). CTFs are nitrogen-rich 2D or 3D polymers and possess high thermal and chemical stability, making them interesting for the fields of gas storage, catalysis, energy storage, and optoelectronics.^23^ However, conventional CTF synthesis is associated with some challenges, particularly due to the harsh reaction conditions that require sealed and evacuated quartz ampules. As an example, an increase in gas pressure due to decomposing monomers or moisture in the ZnCl_2_ must be taken into account. Our group introduced a simpler method, synthesizing CTFs in molten zinc chloride using open ceramic crucibles. A preformed polymer network, synthesized in strong Brønsted acids, undergoes final polymerization and crystallization in molten salt in very short time. This two-step process eliminates the need for specialized equipment, facilitating larger-scale synthesis.^24^ Various other approaches to CTF formation were also presented, from microwave synthesis by using trifluoromethanesulfonic acid as a catalyst^25^ to the use of amidines and aldehydes as monomers,^26^ all of which can lead to CTF synthesis at much lower temperatures and in less aggressive reaction environments. Even the scaling up of CTF synthesis to the large scale was recently reported.^27^

While CTFs do not challenge the assumption that reversible covalent bond formation has to be present to form extended crystalline frameworks, as the reaction conditions chosen are often so harsh that even triazine formation becomes reversible, in recent years, further development in COF chemistry has partially changed this view. Indeed, several COFs have been reported that are directly formed from very stable covalent bonds, where reversibility seems rather unlikely. One example is the formation of vinyl-linked COFs, which from the mechanistical aspect proceeds very similarly to imine formation (replacing the amine by a carbanionic moiety) but yields vinyl bridges that are very stable against, for example, hydrolysis. In 2016, Feng et al. reported the first cyanovinyl-linked COFs via base-catalyzed Knoevenagel condensation between 1,4-phenylacetonitrile and aryl aldehydes,^28^ which was followed after by the group of Jiang et al.^29^ Purely vinylene-linked COFs were reported in 2019 in quick succession, first by the group of Yaghi^30^ and then by us,^31^ Perepichka,^32^ and Zhang.^33^ Notably, such vinylene-linked COFs can undergo 2 + 2 cyclization under irradiation, yielding additional covalent bonds between the layer and thus a 2D to 3D transformation.^31,34^

The “secret ingredients” to prepare these vinylene COFs (V-COFs) are building blocks with acidic C–H protons, which can be easily deprotonated to carbanionic species (which are isoelectronic to amines) and form vinyl bonds by nucleophilic attack of an aldehyde linker. In the first few papers, trimethyltriazine (TMT) was the reagent of choice, possessing relatively acidic protons due to the strong electron accepting property of the triazine ring. This gave an intriguing possibility to prepare COFs from cost-effective monomers. As seen for CTFs, TMT can be formed by acetonitrile trimerization, which gave rise to a one-pot approach to synthesize V-COFs using the commodity chemical acetonitrile as the solvent and reactant, employing a combination of cyclotrimerization and aldol-type condensation reactions with bisaldehydes as a second monomer.^35^ It is therefore not surprising that vinylene-linked COFs are probably the first ones that are produced in kilogram and even larger scales.^36^ Other linkages that have been reported to yield crystalline and highly robust COFs are diazine-,^37^ benzimidazole-,^38^ or dioxin-linked^39^ COFs, which apparently leave no room for reversibility but on the other hand must still be formed under relatively harsh reaction conditions.

A new chapter of COF chemistry with very robust linkages was opened with the introduction of multicomponent reactions (MCRs). MCRs operate in a one-pot cascade manner using at least three components to produce a single product, rendering it an atom-economic synthetic route. MCRs also provide an opportunity to decorate the pores of COFs with different functionalities without the need of postsynthetic modifications. MCRs known in organic synthesis have been used in COF chemistry, including the Doebner, Povarov, Debus–Radziszewski, and Groebke–Blackburn–Bienaymé reactions and a series of novel unnamed MCRs.^40,41^

**Figure 3:**
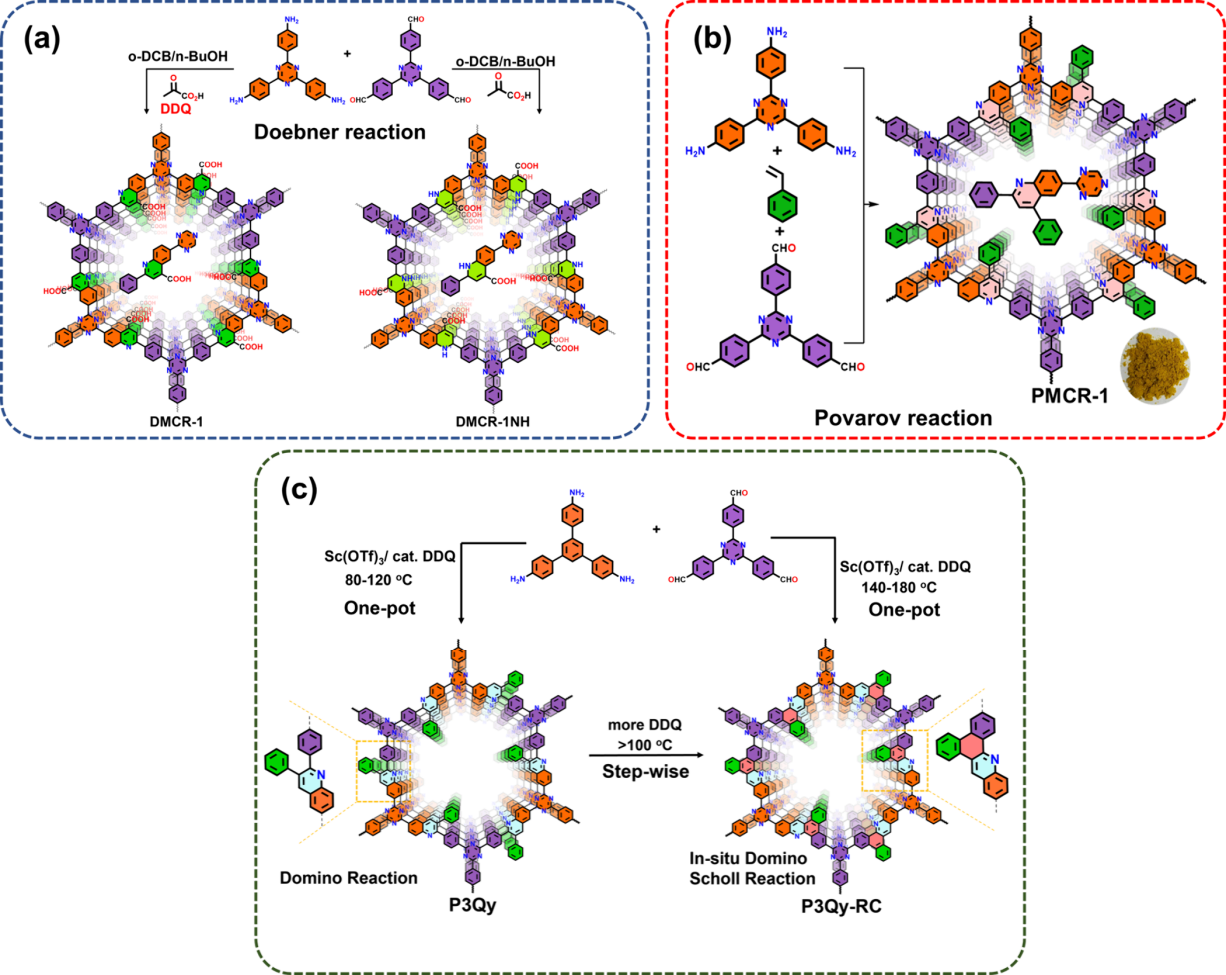
(a) Synthesis scheme for DMCR-1 and DMCR-1NH. Adapted with permission from ref (42). Copyright 2023 American Chemical Society. (b) Synthesis scheme for PMCR-1. Adapted from ref (43). Copyright 2023 Wiley-VCH. (c) Synthesis of heteropolyaromatic MCR-COF. Adapted with permission from ref (4). Copyright 2024 American Chemical Society.

Our laboratory has recently also contributed to the development of MCR COFs. Using the Doebner reaction, we prepared two structurally similar COFs, using amines and aldehyde monomers together with pyruvic acid as a third compound. It was shown that either the dihydro-quinoline-4-carboxylic acid (DMCR-1NH) or the fully aromatic quinoline-4-carboxylic (DMCR-1) acid linkage is formed, the latter when 3-dichloro-5,6-dicyano-1,4-benzoquinone (DDQ) is added as an oxidant (Figure 3a).^42^ The varying degrees of conjugation in the linker significantly affect the optoelectronic properties and photocatalytic performance of the DMCR-COFs. Consequently, these DMCR-COFs demonstrate exceptional hydrogen peroxide (H_2_O_2_) production in pure water, both with and without sacrificial electron donors.

In addition, the multicomponent Povarov reaction can achieve the one-pot synthesis of chemically stable 2D COFs.^44^ By application of aldehyde, amine, and substituted vinyl compounds, the resulting COF displays a unique quinoline-linked structure, with the moiety formerly attached to the vinyl group dangling into the pore (PMCR-1) (Figure 3b). We showed that these MCR-COFs exhibit long-term stable photocatalytic H_2_O_2_ production from pure and seawater.^43^

We recently introduced novel MCR-COFs that are formed from amines, aldehydes, and epoxide instead of vinyl compounds to form unique 2,3-disubtituted quinoline-linked COFs instead of 2,4-substituted ones as observed from the Povarov reaction. Such 2,3-phenylquinoline linkages can undergo a Scholl reaction to form large aromatic linkages in the COF backbone (Figure 3c).^4^ Multicomponent reactions indeed form fully aromatic and chemically robust linkages. However, there are also pathways imaginable in which these robust linkages are formed according to DCC principles if, for example, the imine bonds are initially formed in a reversible manner and the 2 + 4 cyclization with the third compound can only be carried out once a planar configuration in the correct orientation has been achieved.

To summarize this section, the differentiation of whether a linkage is “reversible” or “irreversible” cannot easily be drawn but can rather be used as a description of whether COF linkages can be formed under moderate conditions, yielding however chemically vulnerable bonds, or whether the synthesis requires harsher reaction conditions, yielding robust and stable linkages. However, a strict boundary between these reactions should not be drawn, as transitions are often fluid (consider imine-, β-ketoenamine-, and vinyl-linked COFs) and reversible bonds can also be converted to irreversible ones in situ or by postmodification.

### 2.2 Controlling the Micro- and Macrostructure of 2D COFs

The chemical and nanoscale structures of 2D COFs can be tuned by adjusting the type, geometry, and functionality of the monomers, which determine the backbone chemistry, linkage, pore size, and topology. However, controlling the microstructure and morphology requires additional effort. As COFs are usually obtained as fine powders with pores in the small nanometer range, structural control on the next length scale can indeed be crucial for any desired application. As an example, the micropores in COFs provide their very high surface area but might be hardly accessible for reactants due to diffusion limitations, which raised interest in the fabrication of hierarchical porous COFs. Combining micropores with mesopores and potentially macropores within a single COF lattice holds promise for enhancement of diffusion kinetics. Such a hierarchical porous structure is achievable through hard template-assisted methods widely employed in the fabrication of polymers,^45^ zeolites,^46^ and metal–organic frameworks (MOFs).^47^ These template-assisted strategies involve applying templates (e.g., silica or polymer spheres) which are cast into a matrix and then removed to generate an (additional) porosity, an attempt which has also been recently increasingly used in COFs.^48^

Our group has fabricated crystalline 2D COFs with additional macroporous architectures by templating polystyrene (PS) spheres. This approach has been employed to synthesize various hierarchically porous β-ketoenamine-based COFs. The amount and size of macropores can be easily tuned by the ratio of COF monomers to PS and their size. Using PS templates of varying dimensions showed the perfect replication of the templates, thus fabricating COFs with macropore sizes ranging from ∼160 to ∼360 nm.^49^ The hierarchical porous COFs were applied in electro- and photocatalytic applications and showed in both cases a much higher performance than their purely microporous counterparts, which can be attributed to the enhanced mass transport of molecules to and gaseous products from the catalytic centers.^49^

**Figure 4:**
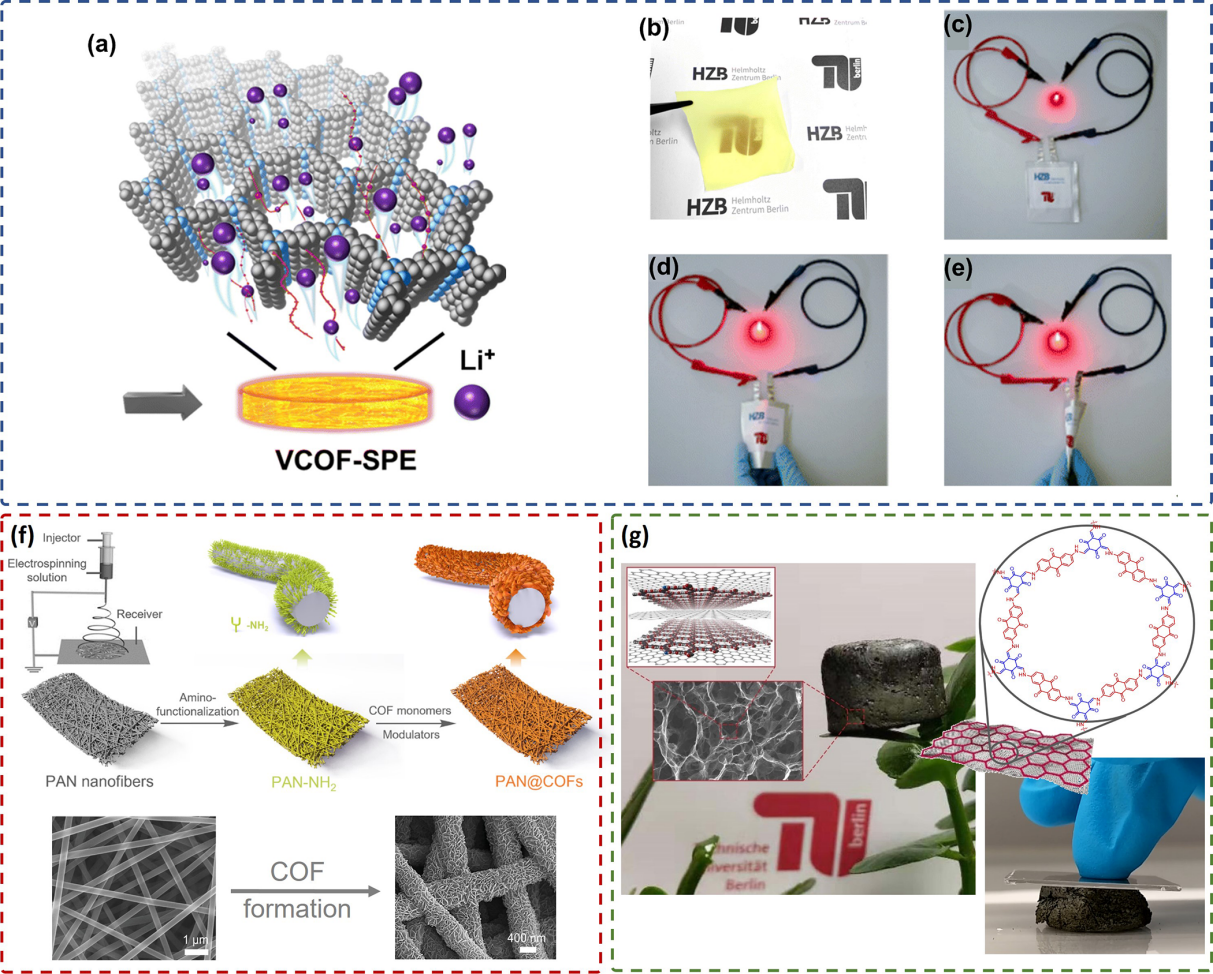
(a) Scheme showing a vinylene-linked COF (V-COF)-based solid polymer electrolyte (SPE); (b) photograph of the prepared V-COF-SPE; (c) abuse tests of the LFP/V-COF-SPE/Li pouch cell after being (d) slightly folded and (e) completely folded. Adapted from ref (50). Copyright 2024 Royal Society of Chemistry. (f) Schematic representation of the in situ growth of COF on amino-functionalized PAN nanofibers prepared by electrospinning. Adapted from ref (51). Copyright 2023 Wiley-VCH. (g) Ultralight COF/rGO aerogel standing on a leaf; inset images show a SEM micrograph of COF/rGO, its chemical structure, and the COF/rGO aerogel under compression. Adapted from ref (1). Copyright 2020 Springer.

Besides small pores, a significant drawback of COFs is that they are usually obtained as very small and undefined particles, while most applications require films, membranes, fibers, granules, monoliths, or other macroscale defined objects. In recent years, many work groups have pioneered the production of COFs in various shapes, such as films, membranes, fibers, or monoliths.^52,53^ In the easiest case, membranes from COFs can be fabricated as mixed matrix membranes; that is, the COF powders are mixed with other polymers, binders, or other required additives to form stable membranes. Such a membrane of a vinylene-linked COF, PEO, PVDF, and LiTFSI has been recently applied as a solid polymer electrolyte (SPE) in Li-ion batteries, preventing Li dendrite growth compared to the COF-free membrane (Figure 4a–e).^50^ COFs can also be grown on a prefabricated substrate, such as a glass slide covered with a transparent conducting oxide (TCO), to produce thin films on electrodes.^54^ Thicker films are possible on structured templates, such as fiber mats. We recently used amine-functionalized fiber mats, prepared from electrospun polyacrylonitrile (PAN) fibers, as a template. 2D imine COFs could be densely grown on the fibers, producing COF membranes of scalable size. Such membranes have been tested for the separation of antibiotics from water (Figure 4f).^51^

The combination of two 2D materials, graphene and 2D COFs, yielded ultralight hierarchically monolithic porous COF/graphene aerogels. The COF chosen is formed in bulk from 1,3,5-triformylphloroglucinol and diaminoanthraquinone through a Schiff-base condensation reaction. However, in this case the monomers were added successively to a graphene oxide (GO) dispersion. The resulting viscous liquid was then transferred to a high-pressure reactor and subjected to heating at 120 °C for 24 h. Following sequential washing steps with water and acetone, a hydrogel was obtained. Upon freeze-drying, COF/rGO (reduced GO) aerogels were obtained and characterized to be 3D, hierarchically porous, and ultralight structures. Notably, in addition to the inherent microporosity of the COF, the resultant aerogels exhibited the formation of large additional macropores (Figure 4g).^1^

In summary, recent years have witnessed innovative methodologies for the synthesis of stable, scalable, and macroscale shaped COFs, an important step toward possible applications.

## 3 Applications of COFs in Photocatalysis

The large surface area, tunable chemical functionality, and especially their π-conjugated structure make COFs interesting materials as photocatalysts. Indeed, COFs have been reported as photocatalysts for various chemical transformations, including hydrogen evolution reaction (HER),^2,3^ overall water splitting,^55^ carbon dioxide reduction reaction (CO_2_RR),^56^ H_2_O_2_ production,^42,43,57^ and organic transformations.^17,18^

Hydrogen is a promising energy carrier, offering a pathway toward sustainable energy solutions. In 2009, polymeric carbon nitride was introduced as a metal-free photocatalyst for hydrogen evolution from water, which sparked interest in organic semiconductors as photocatalysts for this reaction.^58^ At first, conjugated microporous polymers^59^ were reported, followed shortly by the first COFs for photocatalytic HER by Lotsch and co-workers.^60^ We demonstrated that CTF-1 can be applied as a photocatalyst, showing high HER activity under visible light (>420 nm).^61^ Shortly afterward, we presented two β-ketoenamine COFs, with conjugated diacetylene moieties (—C≡C—C≡C—) shown to be crucial for enhancing photochemical water reduction activity.^62^

**Figure 5:**
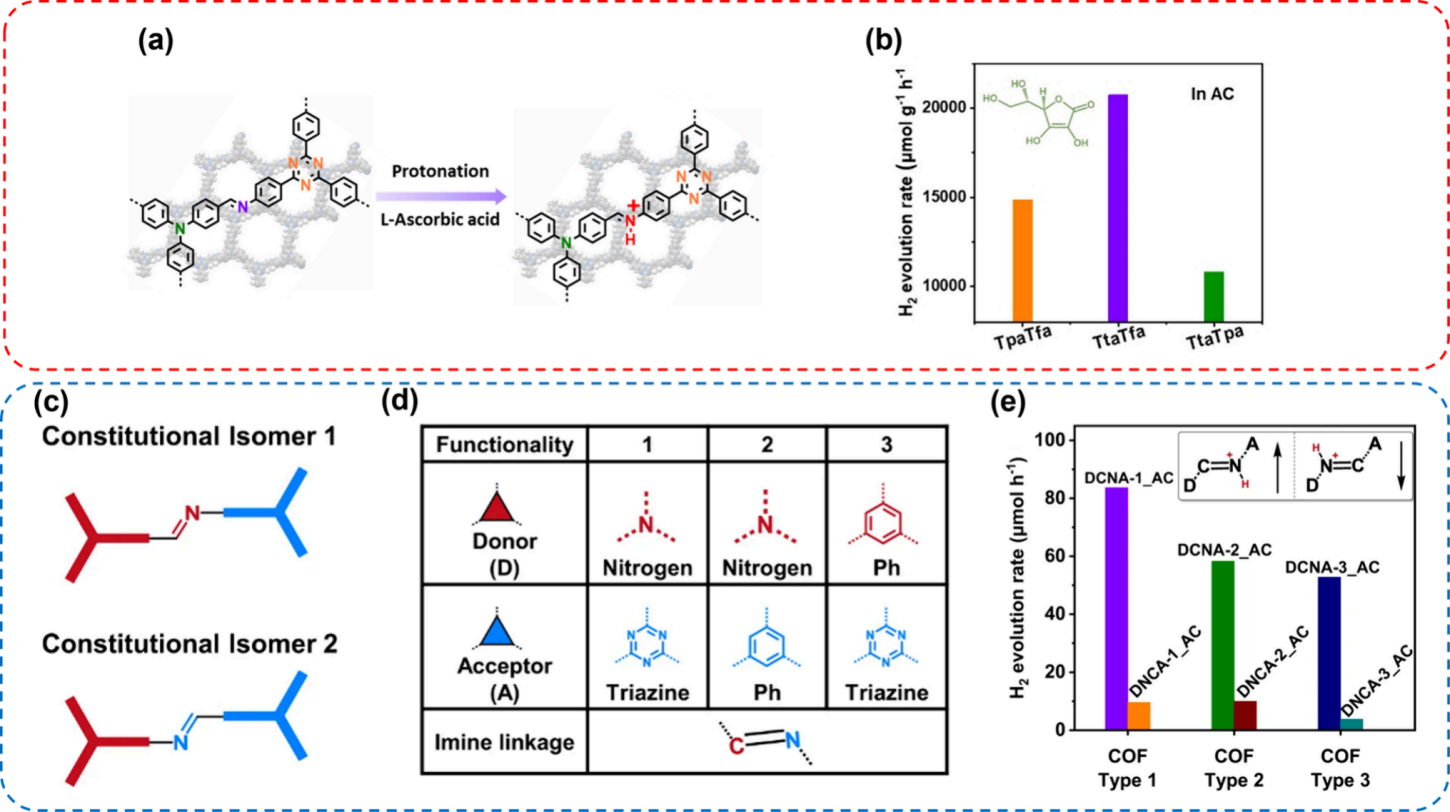
(a) Protonation of an imine-linked COF with l-ascorbic acid and (b) comparison of photocatalytic HER rates of three imine-linked COFs, using ascorbic acid (AC) as the sacrificial electron donor (SED). Adapted from ref (2). Copyright 2021 Wiley-VCH. (c) Scheme of constitutionally isomeric imine-linked donor–acceptor (D-A) COFs; (d) building blocks for the COF synthesis; (e) comparison of the photocatalytic HER rates of the three different COFs. Adapted from ref (3). Copyright 2022 Springer.

It seems obvious that structural characteristics such as the surface area or band gap of COF materials should have a major influence on their photocatalytic performance. This has been verified several times; however, other factors also play an important role, such as polarity and thus wettability of the surfaces, which provides the possibility of water molecules and protons to enter the porous system and access the high surface areas. In 2021, we further showed that the protonation of imine-linked COFs is a crucial step in HER, as it induces significant variations in their (opto)electronic properties, leading to notably enhanced performance in photocatalytic HER in water (Figure 5a). This enhancement is attributed to the improved light absorption capacity, enhanced charge separation efficiency, and higher hydrophilicity of imine-linked COFs upon protonation. In this study, three highly crystalline imine COFs, composed of alternating donor–acceptor (D-A) moieties of varying strengths, were synthesized. Among these COFs, the one featuring the strongest acceptor (triazine) and strongest donor (triphenylamine) moiety demonstrated the highest photocatalytic HER performance, achieving a noteworthy HER rate of 20.7 mmol g^–1^ h^–1^ (Figure 5b).^2^

Several other studies confirmed that electron donor–acceptor pairs in the COF backbone are beneficial to their photocatalytic performance. However, the linkage between these moieties has to also be taken into account. In a recent study, constitutionally isomeric imine-linked donor–acceptor (D-A) COFs were synthesized, featuring distinct orientations of the imine bonds, denoted as DCNA (donor-C=N-acceptor) and DNCA (donor-N=C-acceptor) (Figure 5c,d).^3^ These constitutional isomers displayed notable discrepancies in their photophysical characteristics and consequently in their efficiency as photocatalysts. Specifically, all DCNA COFs exhibited superior performance in photocatalytic HER compared to their DNCA counterparts (Figure 5e). Experimental findings, complemented by density functional theory (DFT) simulations, underscored the pivotal role of imine linkage orientation in governing the band gap, particularly the conduction band minimum (CBM), thereby elucidating the diverse catalytic behaviors observed. Also, the number of hydroxy groups on the 1,3,5-triformylbenzene (Tf) linker, commonly applied for imine or β-ketoenamine COFs, notably influenced the properties of the COF. In COFs with anthracene-containing building blocks, two hydroxy groups on Tf yielded the narrowest band gap (1.8 eV) and efficient exciton migration, resulting in the highest hydrogen evolution rate (8.4 ± 0.5 mmol g^–1^ h^–1^) in powder form. In addition, COF films with increasing hydroxy functionalization showed a steady rise in hydrogen evolution rate, peaking at three hydroxy groups (1.6 ± 0.2 mmol m^–2^ h^–1^) due to enhanced charge carrier mobility.^54^ A current study describes the effect of pore functionality in MCR-COF toward long-term photocatalytic H_2_ production. Phenyl, 2-pyridine, 4-pyridine, imidazole, and pentafluorobenzene pore functional MCR-COFs were synthesized and showed varying performances in photocatalytic HER. As the best in the class, the imidazole-functionalized MCR-COF (PCOF-4) produces 17 mmol g^–1^ h^–1^ of hydrogen using natural sunlight, producing almost 14 L of H_2_ over 8 days in long-term experiments. The photocatalytic HER activity of PCOF-4 can be explained by an interplay of high crystallinity and surface area and improved wettability of the pore’s interior.^63^

**Figure 6:**
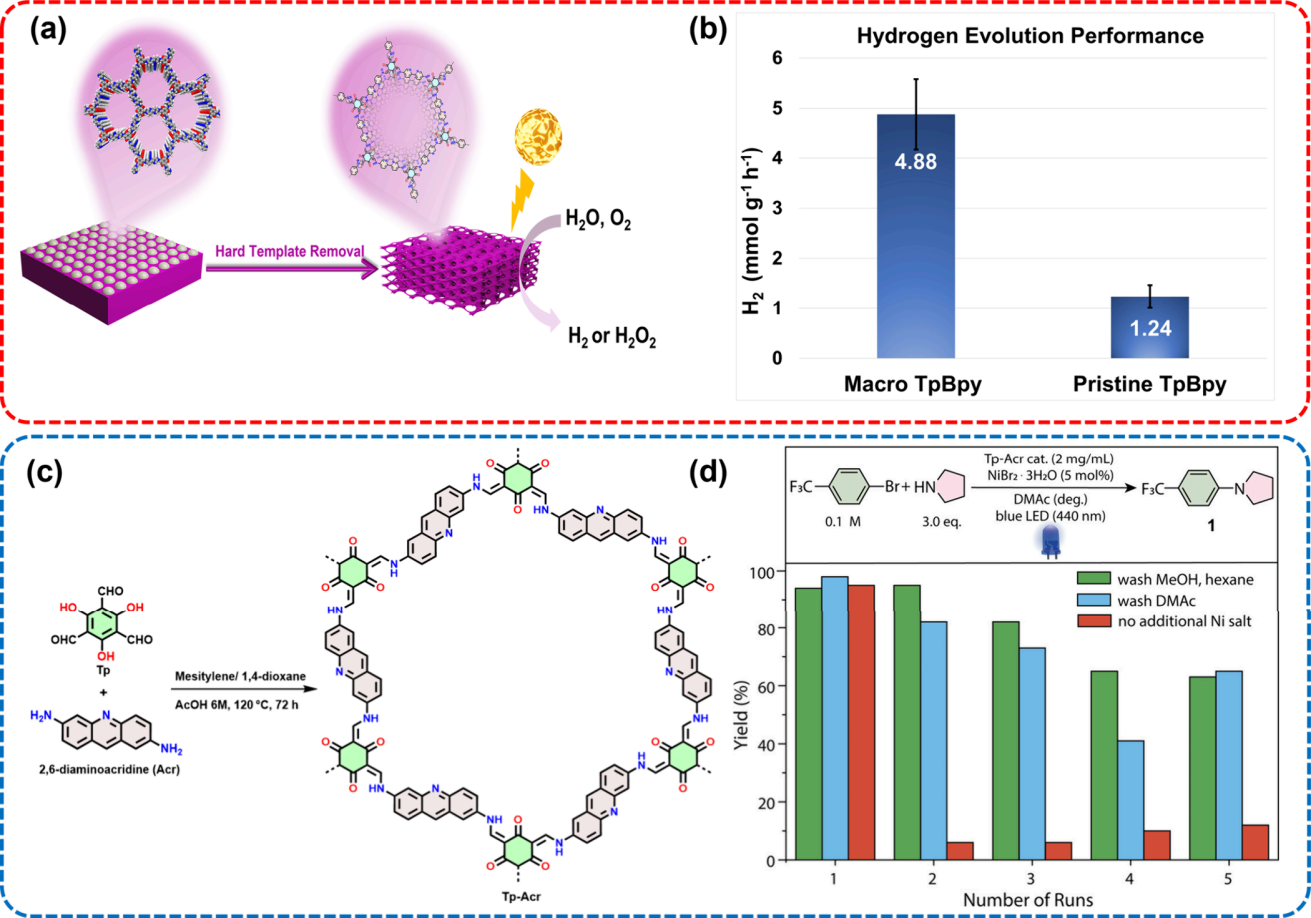
(a) Illustration of the formation of macro TpBpy using PS spheres as template; (b) Photocatalytic HER rates of macro and pristine TpBpy COFs. Adapted from ref (66). Copyright 2024 American Chemical Society (c) Scheme of the synthesis of the acridine-functionalized COFs; (f) Reusability of Tp-Acr COF in the dual nickel/photocatalytic amination of 4-bromobenzotrifluoride and pyrrolidine. Adapted from ref (17). Copyright 2022 Wiley-VCH.

H_2_O_2_ is a pivotal oxidizing agent with applications in wastewater treatment, paper processing, sterilization, chemical synthesis, and fuel cell technologies.^64^ Its global demand has surged to 4 million tons annually, with projections reaching 5.7 million tons by 2027.^65^ Notably, owing to its energy density comparable to that of compressed hydrogen, it has emerged as a promising energy storage medium. We recently prepared the first example of completely metal-free COFs for photocatalytic H_2_O_2_ production, producing 97 and 91 μmol g^–1^ h^–1^ H_2_O_2_, respectively, in a water:ethanol medium through oxygen reduction.^57^ 4-Carboxyl-quinoline (DMCR-1)- or 4-carboxyl-dihydroquinoline (DMCR-1NH)-linked MCR-COFs (Figure 3a) were also shown to be highly suitable for photocatalytic H_2_O_2_ generation.^42,43^ Photocatalytic experiments conducted in water with or without a sacrificial electron donor under air or under O_2_ purging revealed varying rates of H_2_O_2_ production. Under ambient conditions in pure water, H_2_O_2_ yields were determined to be 2264.5 and 1876.3 μmol g^–1^ h^–1^ for DMCR-1NH and DMCR-1, respectively. DMCR-1NH demonstrated superior photocatalytic performance, likely attributable to its more negative conduction band energy and higher hydrophilicity of the surface.^42^ Also, an MCR-COF prepared by the Povarov reaction (PMCR-1) (Figure 3b) facilitated substantial H_2_O_2_ generation in both freshwater and seawater under natural sunlight.^43^ Very recently, our group applied a hierarchical porous COF (denoted as macro TpBpy COF) for photocatalytic HER and H_2_O_2_ production (Figure 6a). Macro TpBpy COF exhibited a HER rate of 4.88 mmol g^–1^ h^–1^, a 4-fold improvement compared to the COF analogue featuring micropores only (Figure 6b). Furthermore, the introduction of macropores proved to be beneficial for the photocatalytic production of H_2_O_2_.^66^

Furthermore, COFs have found applications in photoinduced radical polymerization reactions, namely, the transformation of methyl methacrylate to poly(methyl methacrylate). Significantly, the heterogeneous nature of COF catalysts allowed for facile postreaction separation and subsequent reuse.^67^ In addition, we investigated a series of acridine-functionalized COFs as photocatalysts in metallaphotocatalytic C–N and C–S cross-coupling reactions (Figure 6c, d). The COF was first applied as a photosensitizer together with a molecular Ni-bipyridine catalyst, thus creating a semiheterogeneous catalytic system.^17^ Finally, a fully heterogeneous catalytic system was achieved by fabricating a multicomponent COF, which combined the acridine photosensitizer and the bipyridine ligand within one framework. Several experiments proved that the high activity in metallaphotocatalytic C–S cross-coupling is indeed due to the close proximity of the functional compounds within the backbone.

In summary, the emerging field of COF-based photocatalysis presents distinct advantages over traditional photocatalysts, including adaptability and recyclability. However, research in this area is still in its early stages with various challenges to overcome. Currently, for almost all photocatalytic reduction reactions (HER, CO_2_RR, etc.), a sacrificial oxidant has to be added. This was certainly justified in the early years of COF photocatalysis research to investigate factors that influence and enhance the respective half-reactions. Now, however, it seems to be time to concentrate much more on the other half-reaction, namely photocatalytic water oxidation, for which there are virtually no studies on COFs^55^ but which is crucial to finally create a system that can be used in practice.

## 4 Conclusion

COFs represent a promising class of porous materials for diverse applications. Their remarkable properties include high surface areas characterized by well-defined pores, chemical and thermal stability, and the ability to incorporate tunable functional sites, making them, for example, suitable for catalytic applications.

Various COF-based 2D catalysts have been used for a wide range of chemical reactions. These catalysts often open up reaction pathways that cannot be explored with homogeneous catalysts or other materials, e.g., the coupling of photosensitizers and metal–organic catalysts as the supporting part of the COF backbone.

Several factors are advantageous when designing COFs for specific applications. (i) Predictable structure and composition: the use of reticular chemistry principles in the COF design allows for predictive and precise control over the structure and composition. This predictability aids in exploring structure–activity relationships and catalytic mechanisms, enabling the creation of COFs with specific functionalities tailored for particular applications. (ii) Uniform and tunable pore structures: COFs feature highly uniform and tunable pore sizes, which are crucial for optimizing mass transfer and enhancing the catalytic performance. Adjusting the pore sizes also enables selective size sieving and control over confinement effects, which are essential for facilitating complex chemical transformations. (iii) High surface area: the substantial surface area of COFs results in a large number of easily accessible active sites. This is particularly beneficial for heterogeneous catalysis, where maximizing interactions between reactants and catalysts is essential to improve the reaction efficiency and yield. (iv) Thermal and chemical stability: as entirely covalently connected and often fully π-conjugated materials, COFs exhibit considerable thermal and chemical stability for organic materials. Large progress has been made in recent years to enhance this stability further, allowing them to now perform well in various reaction environments, including those with elevated temperatures or corrosive conditions. This stability ensures consistent catalytic performance across different media, expanding their practical applications. (v) Versatile pore space and composite formation: the customizable pore space in COFs facilitates the incorporation of guest species such as metal nanoparticles or enzymes to create multifunctional composites. This adaptability enhances their catalytic properties and broadens their use in areas such as gas storage, separation, and sensing. (vi) Modular functionalization: COFs can be functionalized with various chemical groups. This allows for the adjustment of properties such as hydrophilicity, electronic behavior, and steric effects, optimizing COFs for specific catalytic reactions or processes. (vii) Potential for sustainable catalysis: COFs align well with green chemistry principles due to their stability, reusability, and efficiency under mild conditions, making them attractive for sustainable catalysis with a reduced environmental impact.

A comprehensive understanding of the structure and properties of COFs paves the way for the development of customized materials with improved performance and applicability in various fields.
